# Supplementary material for: Beyond the ABCs—Discovery of Three New Plasmid Types in Rhodobacterales (RepQ, RepY, RepW)
Source: Microorganisms. 2022 Mar 29;10(4):738. doi: 10.3390/microorganisms10040738 (PMC9025767; doi:10.3390/microorganisms10040738)

Mapping of PacBio and Illumina reads on replicons of *Sulfitobacter dubius* DSM 109990

A) Mapping of PacBio (top) and Illumina (bottom) reads on cDSM109990 (1-25 kb)

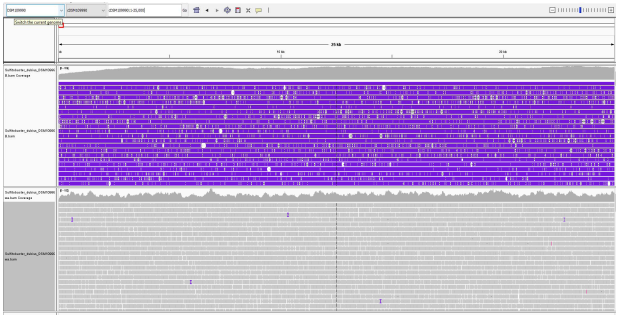

B) Mapping of PacBio (top) and Illumina (bottom) reads on pDSM109990_a (1-25 kb)

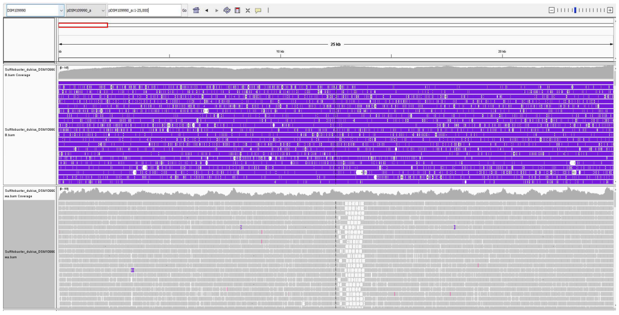

C) Mapping of PacBio (top) and Illumina (bottom) reads on pDSM109990_b (1-25 kb)

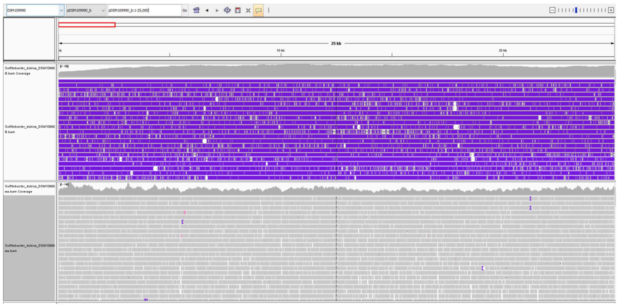

D) Mapping of PacBio (top) and Illumina (bottom) reads on pDSM109990_c (1-25 kb)

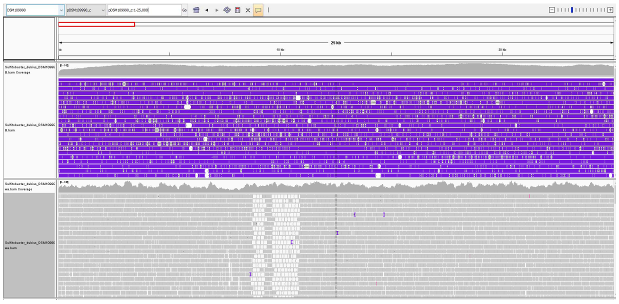

E) Mapping of PacBio (top) and Illumina (bottom) reads on pDSM109990_d (1-25 kb)

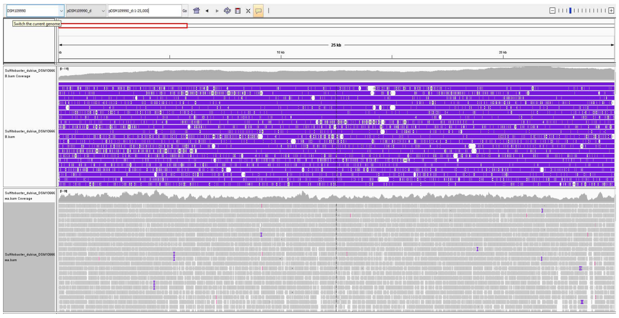

F) Mapping of PacBio (top) and Illumina (bottom) reads on pDSM109990_e (1-25 kb)

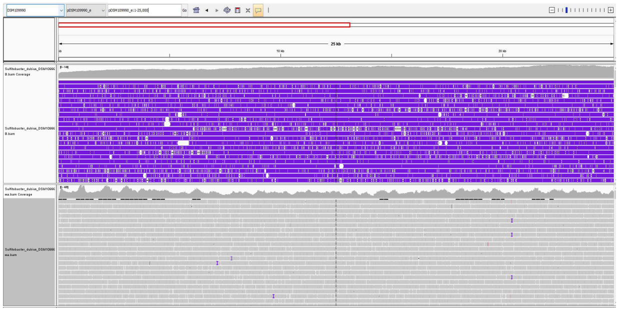

G) Mapping of PacBio (top) and Illumina (bottom) reads on pDSM109990_f

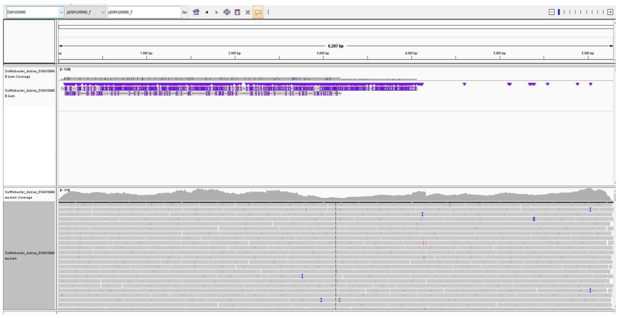

H) Mapping of PacBio (top) and Illumina (bottom) reads on pDSM109990_g

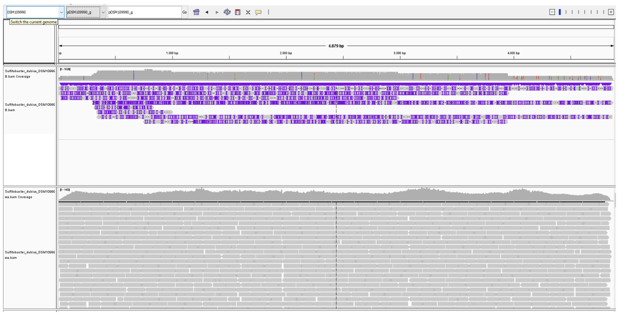

I) Mapping of PacBio (top) and Illumina (bottom) reads on pDSM109990_h

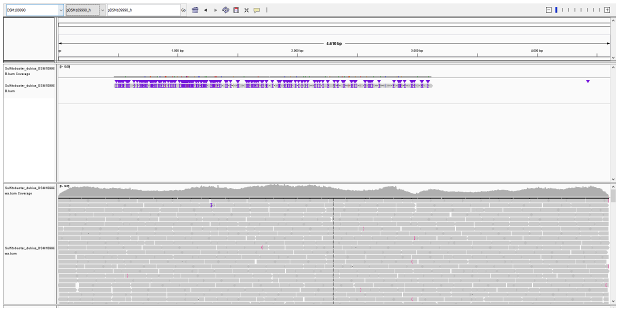

J) Mapping of PacBio (top) and Illumina (bottom) reads on pDSM109990_i

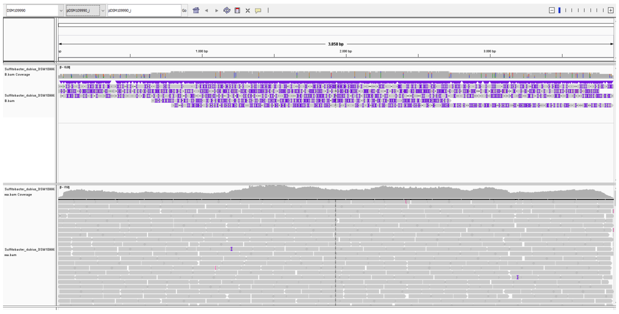

Supplement: Supplementary file 1 [file microorganisms-10-00738-s001.zip › Supplementary Figures & Tables/Figure_S1_Read_mapping_DSM109990_mod_211102.pdf]
