# Supplementary material for: Beyond the ABCs—Discovery of Three New Plasmid Types in Rhodobacterales (RepQ, RepY, RepW)
Source: Microorganisms. 2022 Mar 29;10(4):738. doi: 10.3390/microorganisms10040738 (PMC9025767; doi:10.3390/microorganisms10040738)
Supplement: Supplementary file 1 [file microorganisms-10-00738-s001.zip › Supplementary Figures & Tables/Figure_S3new_dendrograms_cu_tetra_man_220225.pdf]

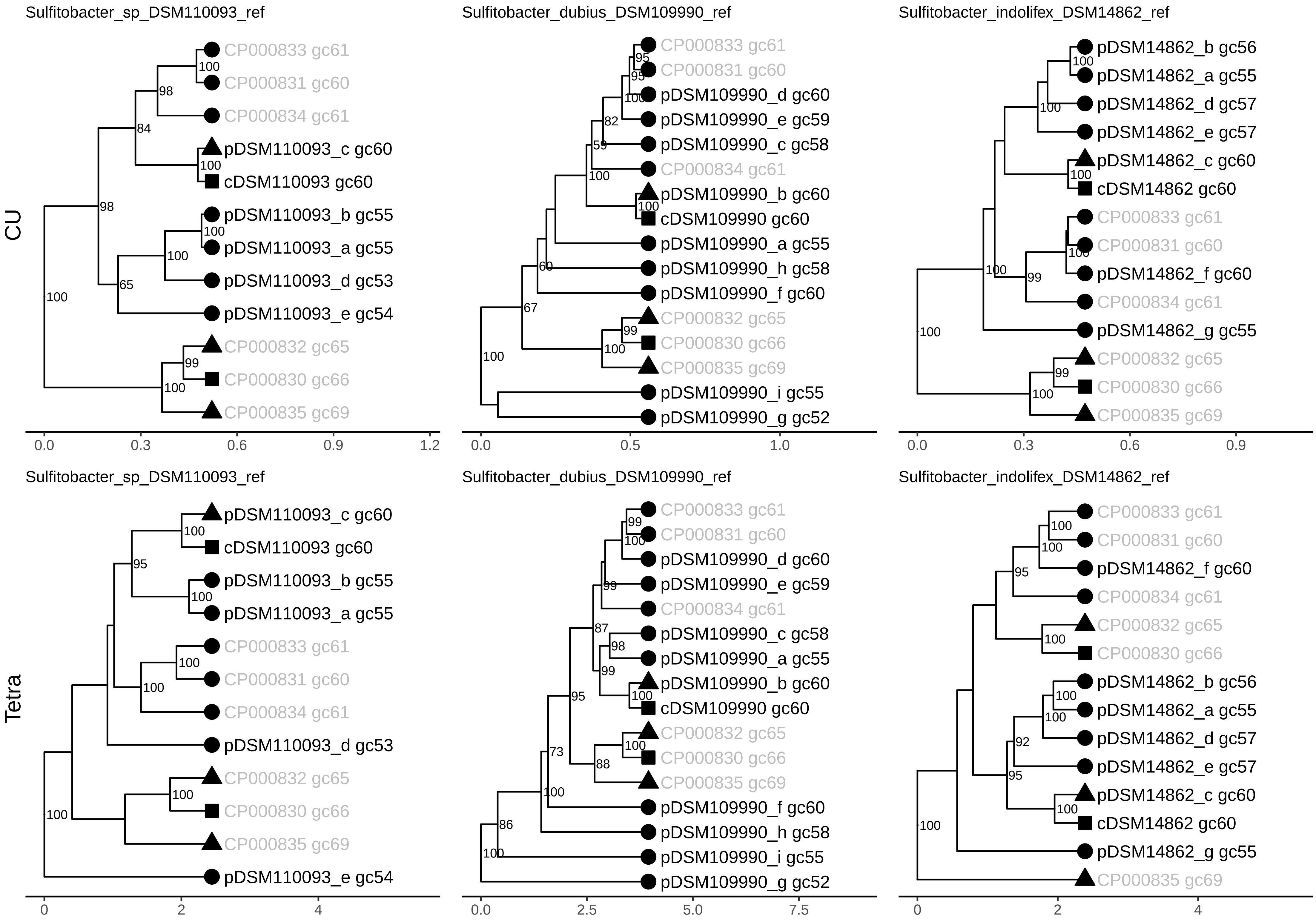
CU
Tetra
Sulfitobacter_sp_DSM110093_ref
CP000833 gc61
CP000831 gc60
CP000834 gc61
pDSM110093_c gc60
cDSM110093 gc60
pDSM110093_b gc55
pDSM110093_a gc55
pDSM110093_d gc53
pDSM110093_e gc54
CP000832 gc65
CP000830 gc66
CP000835 gc69
0.0 0.3 0.6 0.9 1.2
Sulfitobacter_dubius_DSM109990_ref
CP000833 gc61
CP000831 gc60
pDSM109990_d gc60
pDSM109990_e gc59
pDSM109990_c gc58
CP000834 gc61
pDSM109990_b gc60
cDSM109990 gc60
pDSM109990_a gc55
pDSM109990_h gc58
pDSM109990_f gc60
CP000832 gc65
CP000830 gc66
CP000835 gc69
pDSM109990_i gc55
pDSM109990_g gc52
0.0 0.5 1.0
Sulfitobacter_indolifex_DSM14862_ref
pDSM14862_b gc56
pDSM14862_a gc55
pDSM14862_d gc57
pDSM14862_e gc57
pDSM14862_c gc60
cDSM14862 gc60
CP000833 gc61
CP000831 gc60
pDSM14862_f gc60
CP000834 gc61
pDSM14862_g gc55
CP000832 gc65
CP000830 gc66
CP000835 gc69
0.0 0.3 0.6 0.9
Sulfitobacter_sp_DSM110093_ref
pDSM110093_c gc60
cDSM110093 gc60
pDSM110093_b gc55
pDSM110093_a gc55
CP000833 gc61
CP000831 gc60
CP000834 gc61
pDSM110093_d gc53
CP000832 gc65
CP000830 gc66
CP000835 gc69
pDSM110093_e gc54
0 2 4
Sulfitobacter_dubius_DSM109990_ref
CP000833 gc61
CP000831 gc60
pDSM109990_d gc60
pDSM109990_e gc59
CP000834 gc61
pDSM109990_c gc58
pDSM109990_a gc55
pDSM109990_b gc60
cDSM109990 gc60
CP000832 gc65
CP000830 gc66
CP000835 gc69
pDSM109990_f gc60
pDSM109990_h gc58
pDSM109990_i gc55
pDSM109990_g gc52
0.0 2.5 5.0 7.5
Sulfitobacter_indolifex_DSM14862_ref
CP000833 gc61
CP000831 gc60
pDSM14862_f gc60
CP000834 gc61
CP000832 gc65
CP000830 gc66
pDSM14862_b gc56
pDSM14862_a gc55
pDSM14862_d gc57
pDSM14862_e gc57
pDSM14862_c gc60
cDSM14862 gc60
pDSM14862_g gc55
CP000835 gc69
0 2 4

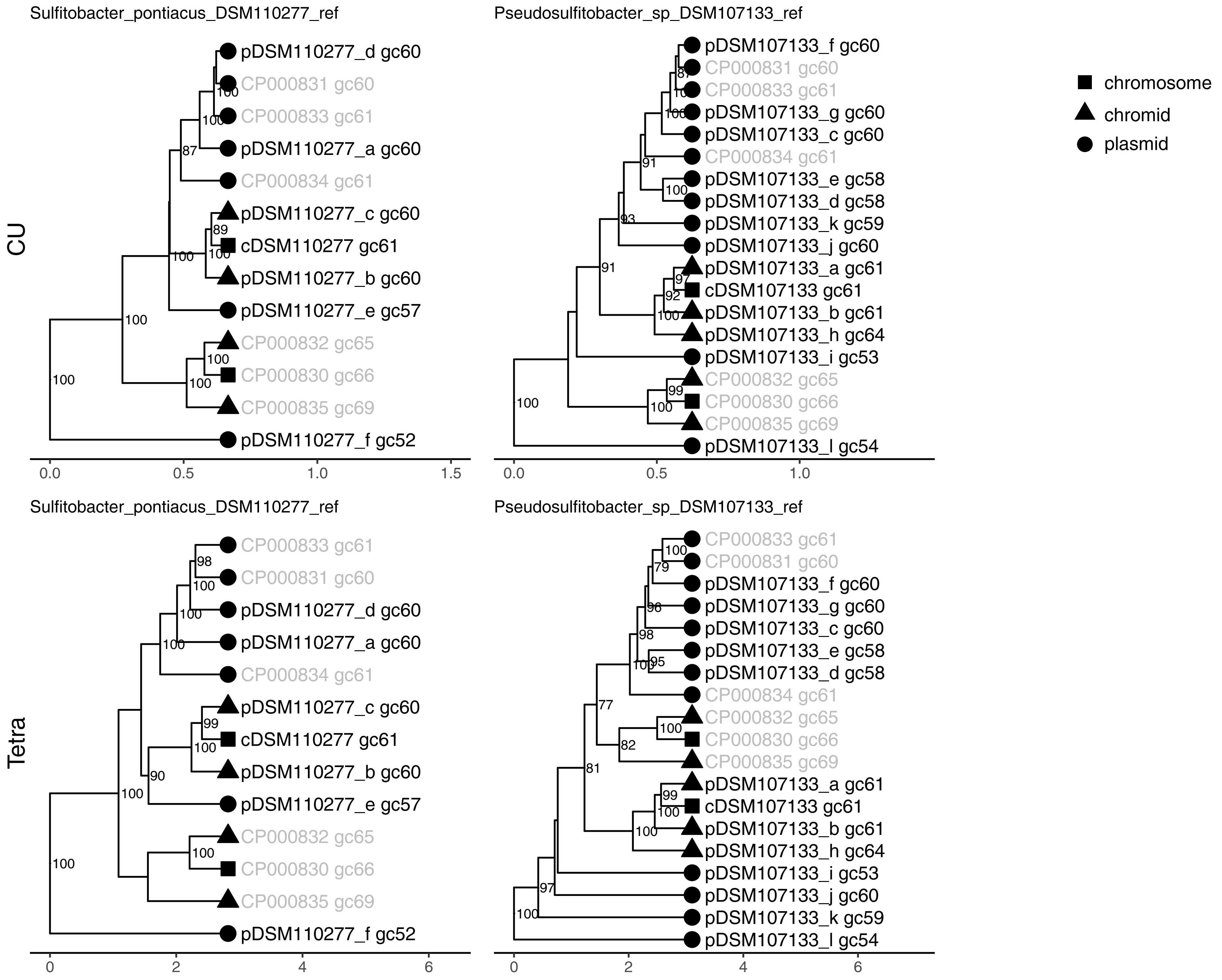

CU
Tetra
Sulfitobacter_pontiacus_DSM110277_ref
Pseudosulfitobacter_sp_DSM107133_ref
pDSM110277_d gc60
CP000831 gc60
CP000833 gc61
pDSM110277_a gc60
CP000834 gc61
pDSM110277_c gc60
cDSM110277 gc61
pDSM110277_b gc60
pDSM110277_e gc57
CP000832 gc65
CP000830 gc66
CP000835 gc69
pDSM110277_f gc52
pDSM107133_f gc60
pDSM107133_g gc60
pDSM107133_c gc60
pDSM107133_e gc58
pDSM107133_d gc58
pDSM107133_k gc59
pDSM107133_j gc60
pDSM107133_a gc61
cDSM107133 gc61
pDSM107133_b gc61
pDSM107133_h gc64
pDSM107133_i gc53
pDSM107133_l gc54
chromosome
chromid
plasmid
