# Supplementary material for: Beyond the ABCs—Discovery of Three New Plasmid Types in Rhodobacterales (RepQ, RepY, RepW)
Source: Microorganisms. 2022 Mar 29;10(4):738. doi: 10.3390/microorganisms10040738 (PMC9025767; doi:10.3390/microorganisms10040738)
Supplement: Supplementary file 1 [file microorganisms-10-00738-s001.zip › Supplementary Figures & Tables/Figure_S6new_Phylogeny-Complete_RepQ_211010.pptx]

## Slide 1
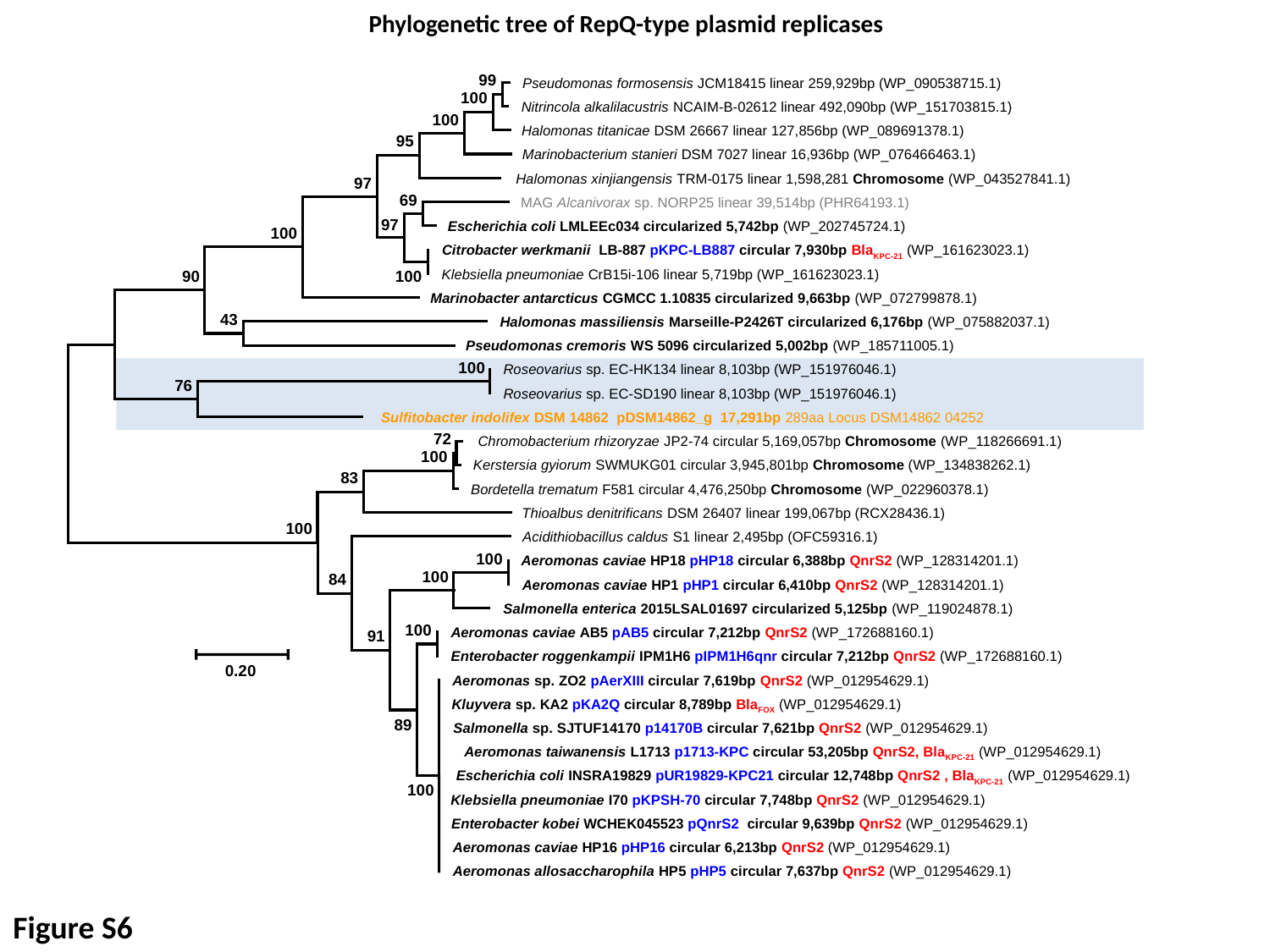

Phylogenetic tree of RepQ-type plasmid replicases
99
100
100
95
97
69
97
100
90
100
43
100
76
72
100
83
100
100
100
84
100
91
89
100
0.20
 Pseudomonas formosensis JCM18415 linear 259,929bp (WP_090538715.1)
 Nitrincola alkalilacustris NCAIM-B-02612 linear 492,090bp (WP_151703815.1)
 Halomonas titanicae DSM 26667 linear 127,856bp (WP_089691378.1)
 Marinobacterium stanieri DSM 7027 linear 16,936bp (WP_076466463.1)
 Halomonas xinjiangensis TRM-0175 linear 1,598,281 Chromosome (WP_043527841.1)
 MAG Alcanivorax sp. NORP25 linear 39,514bp (PHR64193.1)
 Escherichia coli LMLEEc034 circularized 5,742bp (WP_202745724.1)
 Citrobacter werkmanii LB-887 pKPC-LB887 circular 7,930bp BlaKPC-21 (WP_161623023.1)
 Klebsiella pneumoniae CrB15i-106 linear 5,719bp (WP_161623023.1)
 Marinobacter antarcticus CGMCC 1.10835 circularized 9,663bp (WP_072799878.1)
 Halomonas massiliensis Marseille-P2426T circularized 6,176bp (WP_075882037.1)
 Pseudomonas cremoris WS 5096 circularized 5,002bp (WP_185711005.1)
 Roseovarius sp. EC-HK134 linear 8,103bp (WP_151976046.1)
 Roseovarius sp. EC-SD190 linear 8,103bp (WP_151976046.1)
 Chromobacterium rhizoryzae JP2-74 circular 5,169,057bp Chromosome (WP_118266691.1)
 Kerstersia gyiorum SWMUKG01 circular 3,945,801bp Chromosome (WP_134838262.1)
 Bordetella trematum F581 circular 4,476,250bp Chromosome (WP_022960378.1)
 Thioalbus denitrificans DSM 26407 linear 199,067bp (RCX28436.1)
 Acidithiobacillus caldus S1 linear 2,495bp (OFC59316.1)
 Aeromonas caviae HP18 pHP18 circular 6,388bp QnrS2 (WP_128314201.1)
 Aeromonas caviae HP1 pHP1 circular 6,410bp QnrS2 (WP_128314201.1)
 Salmonella enterica 2015LSAL01697 circularized 5,125bp (WP_119024878.1)
 Aeromonas caviae AB5 pAB5 circular 7,212bp QnrS2 (WP_172688160.1)
 Enterobacter roggenkampii IPM1H6 pIPM1H6qnr circular 7,212bp QnrS2 (WP_172688160.1)
 Aeromonas sp. ZO2 pAerXIII circular 7,619bp QnrS2 (WP_012954629.1)
 Kluyvera sp. KA2 pKA2Q circular 8,789bp BlaFOX (WP_012954629.1)
 Salmonella sp. SJTUF14170 p14170B circular 7,621bp QnrS2 (WP_012954629.1)
 Aeromonas taiwanensis L1713 p1713-KPC circular 53,205bp QnrS2, BlaKPC-21 (WP_012954629.1)
 Escherichia coli INSRA19829 pUR19829-KPC21 circular 12,748bp QnrS2 , BlaKPC-21 (WP_012954629.1)
 Klebsiella pneumoniae I70 pKPSH-70 circular 7,748bp QnrS2 (WP_012954629.1)
 Enterobacter kobei WCHEK045523 pQnrS2 circular 9,639bp QnrS2 (WP_012954629.1)
 Aeromonas caviae HP16 pHP16 circular 6,213bp QnrS2 (WP_012954629.1)
 Aeromonas allosaccharophila HP5 pHP5 circular 7,637bp QnrS2 (WP_012954629.1)
Figure S6
