# Supplementary material for: Beyond the ABCs—Discovery of Three New Plasmid Types in Rhodobacterales (RepQ, RepY, RepW)
Source: Microorganisms. 2022 Mar 29;10(4):738. doi: 10.3390/microorganisms10040738 (PMC9025767; doi:10.3390/microorganisms10040738)
Supplement: Supplementary file 1 [file microorganisms-10-00738-s001.zip › Supplementary Figures & Tables/Figure_S7new_Phylogeny_RepW_61-Taxa_220225.pptx]

## Slide 1
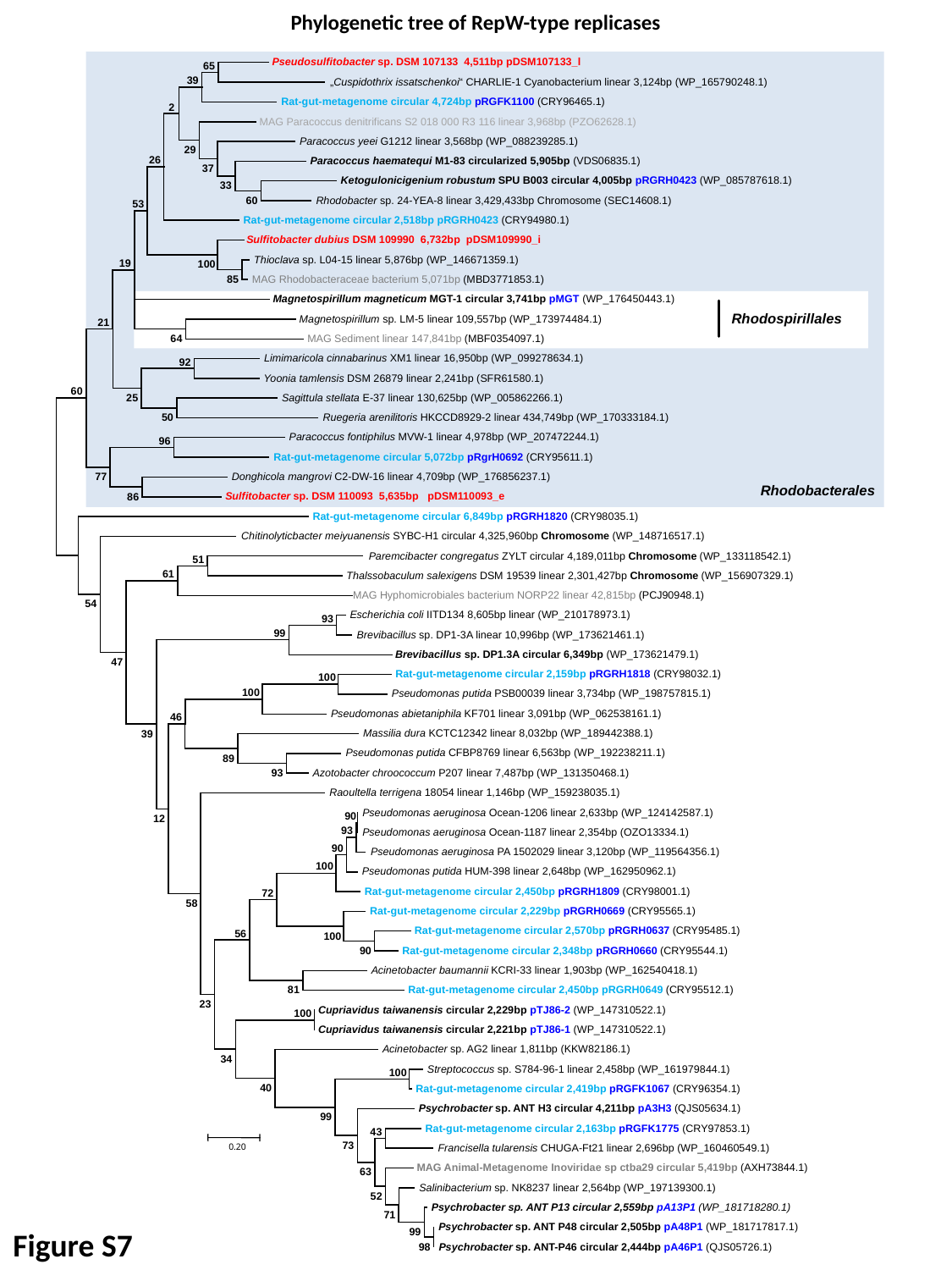

Phylogenetic tree of RepW-type replicases
 Pseudosulfitobacter sp. DSM 107133 4,511bp pDSM107133_l
65
39
2
29
26
37
33
60
53
19
100
85
21
64
92
60
25
50
96
77
86
51
61
54
93
99
47
100
100
46
39
89
93
90
12
93
90
100
72
58
56
100
90
81
23
100
34
100
40
99
43
73
63
52
71
99
98
 „Cuspidothrix issatschenkoi“ CHARLIE-1 Cyanobacterium linear 3,124bp (WP_165790248.1)
 Rat-gut-metagenome circular 4,724bp pRGFK1100 (CRY96465.1)
 MAG Paracoccus denitrificans S2 018 000 R3 116 linear 3,968bp (PZO62628.1)
 Paracoccus yeei G1212 linear 3,568bp (WP_088239285.1)
 Paracoccus haematequi M1-83 circularized 5,905bp (VDS06835.1)
 Ketogulonicigenium robustum SPU B003 circular 4,005bp pRGRH0423 (WP_085787618.1)
 Rhodobacter sp. 24-YEA-8 linear 3,429,433bp Chromosome (SEC14608.1)
 Rat-gut-metagenome circular 2,518bp pRGRH0423 (CRY94980.1)
 Sulfitobacter dubius DSM 109990 6,732bp pDSM109990_i
 Thioclava sp. L04-15 linear 5,876bp (WP_146671359.1)
 MAG Rhodobacteraceae bacterium 5,071bp (MBD3771853.1)
 Magnetospirillum magneticum MGT-1 circular 3,741bp pMGT (WP_176450443.1)
 Rhodospirillales
 Magnetospirillum sp. LM-5 linear 109,557bp (WP_173974484.1)
 MAG Sediment linear 147,841bp (MBF0354097.1)
 Limimaricola cinnabarinus XM1 linear 16,950bp (WP_099278634.1)
 Yoonia tamlensis DSM 26879 linear 2,241bp (SFR61580.1)
 Sagittula stellata E-37 linear 130,625bp (WP_005862266.1)
 Ruegeria arenilitoris HKCCD8929-2 linear 434,749bp (WP_170333184.1)
 Paracoccus fontiphilus MVW-1 linear 4,978bp (WP_207472244.1)
 Rat-gut-metagenome circular 5,072bp pRgrH0692 (CRY95611.1)
 Donghicola mangrovi C2-DW-16 linear 4,709bp (WP_176856237.1)
 Rhodobacterales
 Sulfitobacter sp. DSM 110093 5,635bp pDSM110093_e
 Rat-gut-metagenome circular 6,849bp pRGRH1820 (CRY98035.1)
 Chitinolyticbacter meiyuanensis SYBC-H1 circular 4,325,960bp Chromosome (WP_148716517.1)
 Paremcibacter congregatus ZYLT circular 4,189,011bp Chromosome (WP_133118542.1)
 Thalssobaculum salexigens DSM 19539 linear 2,301,427bp Chromosome (WP_156907329.1)
MAG Hyphomicrobiales bacterium NORP22 linear 42,815bp (PCJ90948.1)
 Escherichia coli IITD134 8,605bp linear (WP_210178973.1)
 Brevibacillus sp. DP1-3A linear 10,996bp (WP_173621461.1)
 Brevibacillus sp. DP1.3A circular 6,349bp (WP_173621479.1)
 Rat-gut-metagenome circular 2,159bp pRGRH1818 (CRY98032.1)
 Pseudomonas putida PSB00039 linear 3,734bp (WP_198757815.1)
 Pseudomonas abietaniphila KF701 linear 3,091bp (WP_062538161.1)
 Massilia dura KCTC12342 linear 8,032bp (WP_189442388.1)
 Pseudomonas putida CFBP8769 linear 6,563bp (WP_192238211.1)
 Azotobacter chroococcum P207 linear 7,487bp (WP_131350468.1)
 Raoultella terrigena 18054 linear 1,146bp (WP_159238035.1)
 Pseudomonas aeruginosa Ocean-1206 linear 2,633bp (WP_124142587.1)
 Pseudomonas aeruginosa Ocean-1187 linear 2,354bp (OZO13334.1)
 Pseudomonas aeruginosa PA 1502029 linear 3,120bp (WP_119564356.1)
 Pseudomonas putida HUM-398 linear 2,648bp (WP_162950962.1)
 Rat-gut-metagenome circular 2,450bp pRGRH1809 (CRY98001.1)
 Rat-gut-metagenome circular 2,229bp pRGRH0669 (CRY95565.1)
 Rat-gut-metagenome circular 2,570bp pRGRH0637 (CRY95485.1)
 Rat-gut-metagenome circular 2,348bp pRGRH0660 (CRY95544.1)
 Acinetobacter baumannii KCRI-33 linear 1,903bp (WP_162540418.1)
 Rat-gut-metagenome circular 2,450bp pRGRH0649 (CRY95512.1)
 Cupriavidus taiwanensis circular 2,229bp pTJ86-2 (WP_147310522.1)
 Cupriavidus taiwanensis circular 2,221bp pTJ86-1 (WP_147310522.1)
 Acinetobacter sp. AG2 linear 1,811bp (KKW82186.1)
 Streptococcus sp. S784-96-1 linear 2,458bp (WP_161979844.1)
 Rat-gut-metagenome circular 2,419bp pRGFK1067 (CRY96354.1)
 Psychrobacter sp. ANT H3 circular 4,211bp pA3H3 (QJS05634.1)
 Rat-gut-metagenome circular 2,163bp pRGFK1775 (CRY97853.1)
0.20
 Francisella tularensis CHUGA-Ft21 linear 2,696bp (WP_160460549.1)
 MAG Animal-Metagenome Inoviridae sp ctba29 circular 5,419bp (AXH73844.1)
 Salinibacterium sp. NK8237 linear 2,564bp (WP_197139300.1)
 Psychrobacter sp. ANT P13 circular 2,559bp pA13P1 (WP_181718280.1)
Figure S7
 Psychrobacter sp. ANT P48 circular 2,505bp pA48P1 (WP_181717817.1)
 Psychrobacter sp. ANT-P46 circular 2,444bp pA46P1 (QJS05726.1)
