# Supplementary material for: Beyond the ABCs—Discovery of Three New Plasmid Types in Rhodobacterales (RepQ, RepY, RepW)
Source: Microorganisms. 2022 Mar 29;10(4):738. doi: 10.3390/microorganisms10040738 (PMC9025767; doi:10.3390/microorganisms10040738)
Supplement: Supplementary file 1 [file microorganisms-10-00738-s001.zip › Supplementary Figures & Tables/Supplementary Figures (PDF)/Figure_S5_Plasmid-Functionality-Test_RepQ-RepY-RepW_220229.pdf]

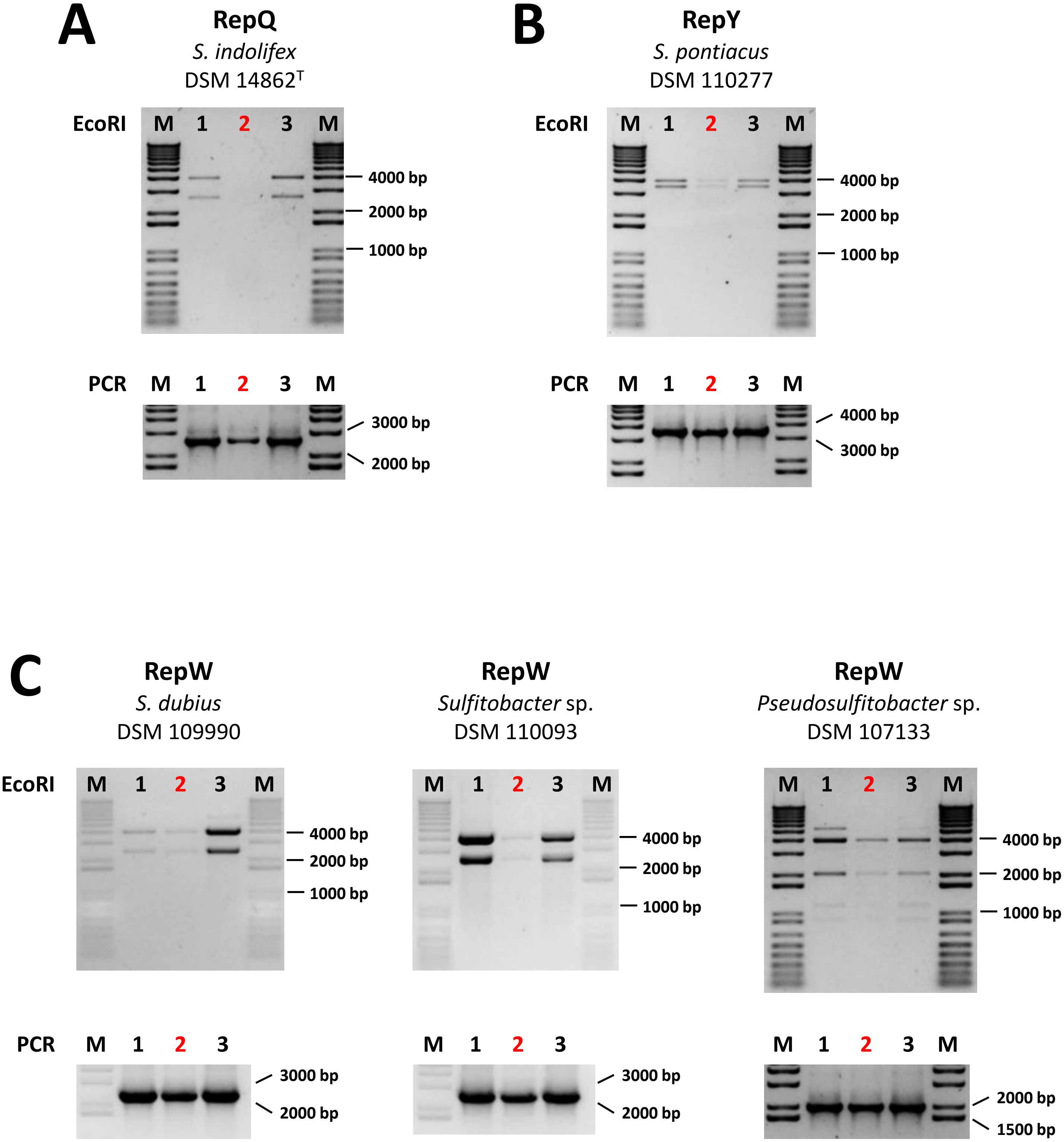
A
RepQ
S. indolifex
DSM 14862T
EcoRI
M 1 2 3 M
4000 bp
2000 bp
1000 bp
PCR
M 1 2 3 M
3000 bp
2000 bp
B
RepY
S. pontiacus
DSM 110277
EcoRI
M 1 2 3 M
4000 bp
2000 bp
1000 bp
PCR
M 1 2 3 M
4000 bp
3000 bp
C
RepW
S. dubius
DSM 109990
EcoRI
M 1 2 3 M
4000 bp
2000 bp
1000 bp
PCR
M 1 2 3
3000 bp
2000 bp
RepW
Sulfitobacter sp.
DSM 110093
M 1 2 3 M
4000 bp
2000 bp
1000 bp
M 1 2 3
3000 bp
2000 bp
RepW
Pseudosulfitobacter sp.
DSM 107133
M 1 2 3 M
4000 bp
2000 bp
1000 bp
M 1 2 3 M
2000 bp
1500 bp
