# Supplementary figures and images for: Beyond the ABCs—Discovery of Three New Plasmid Types in Rhodobacterales (RepQ, RepY, RepW)
Source: Microorganisms. 2022 Mar 29;10(4):738. doi: 10.3390/microorganisms10040738 (PMC9025767; doi:10.3390/microorganisms10040738)

## Phylogenomic tree

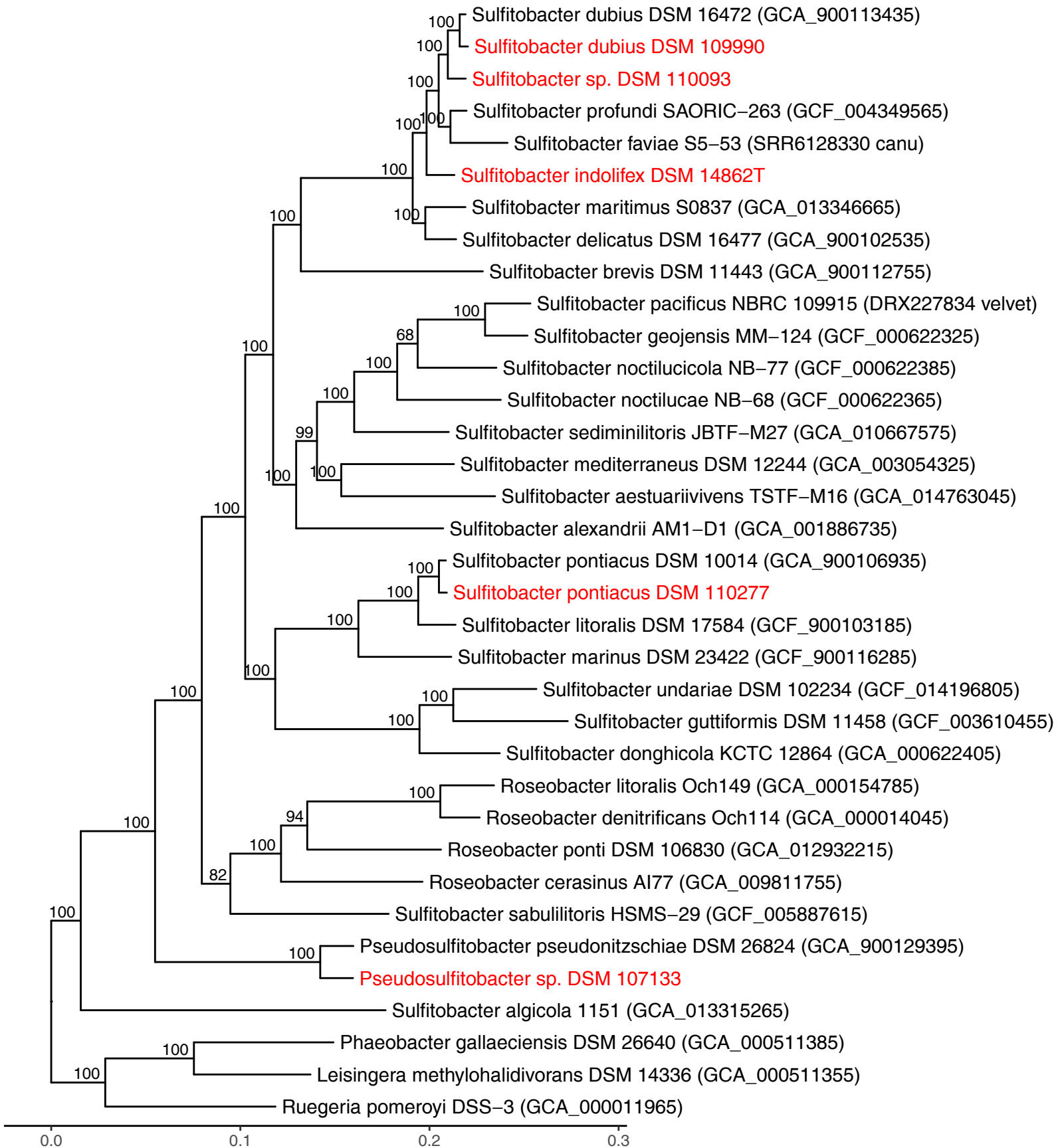

Supplement: Supplementary file 1 [file microorganisms-10-00738-s001.zip › Supplementary Figures & Tables/Figure_S2new_sulfito_type_cryptic_UBCGtree_220225.pdf]

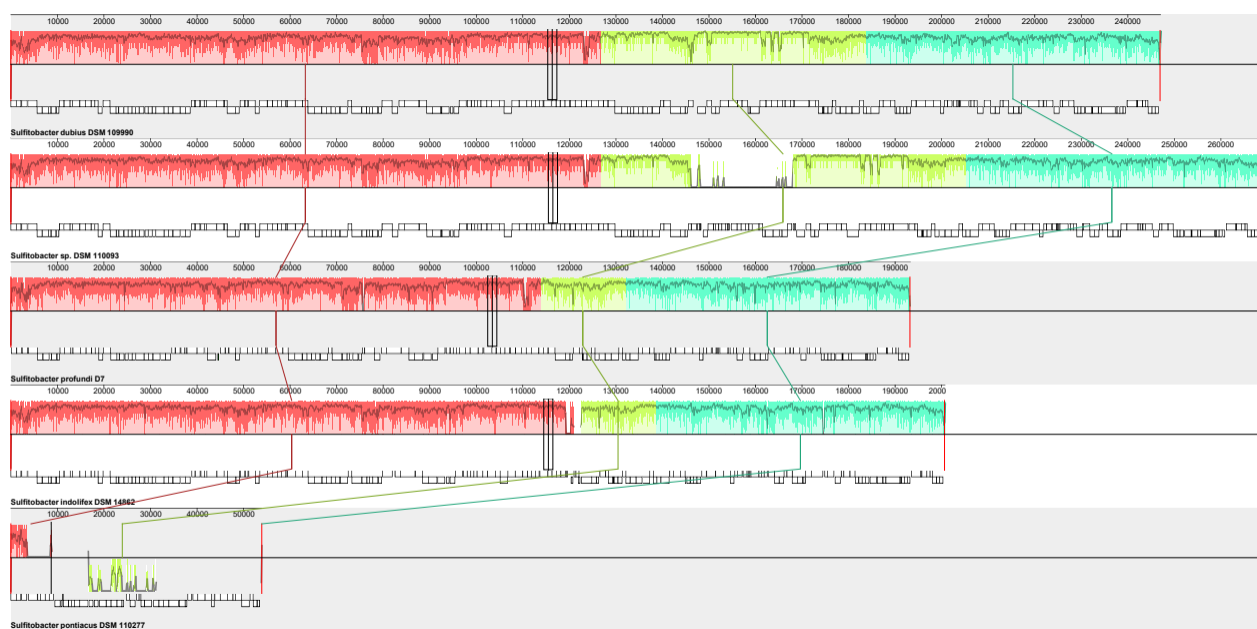

Supplement: Supplementary file 1 [file microorganisms-10-00738-s001.zip › Supplementary Figures & Tables/Figure_S4new_Sulfito_repABC-8_mauve_with_pontiacus_sort_220125.pdf]

# Phylogenetic tree of RepQ-type plasmid replicases

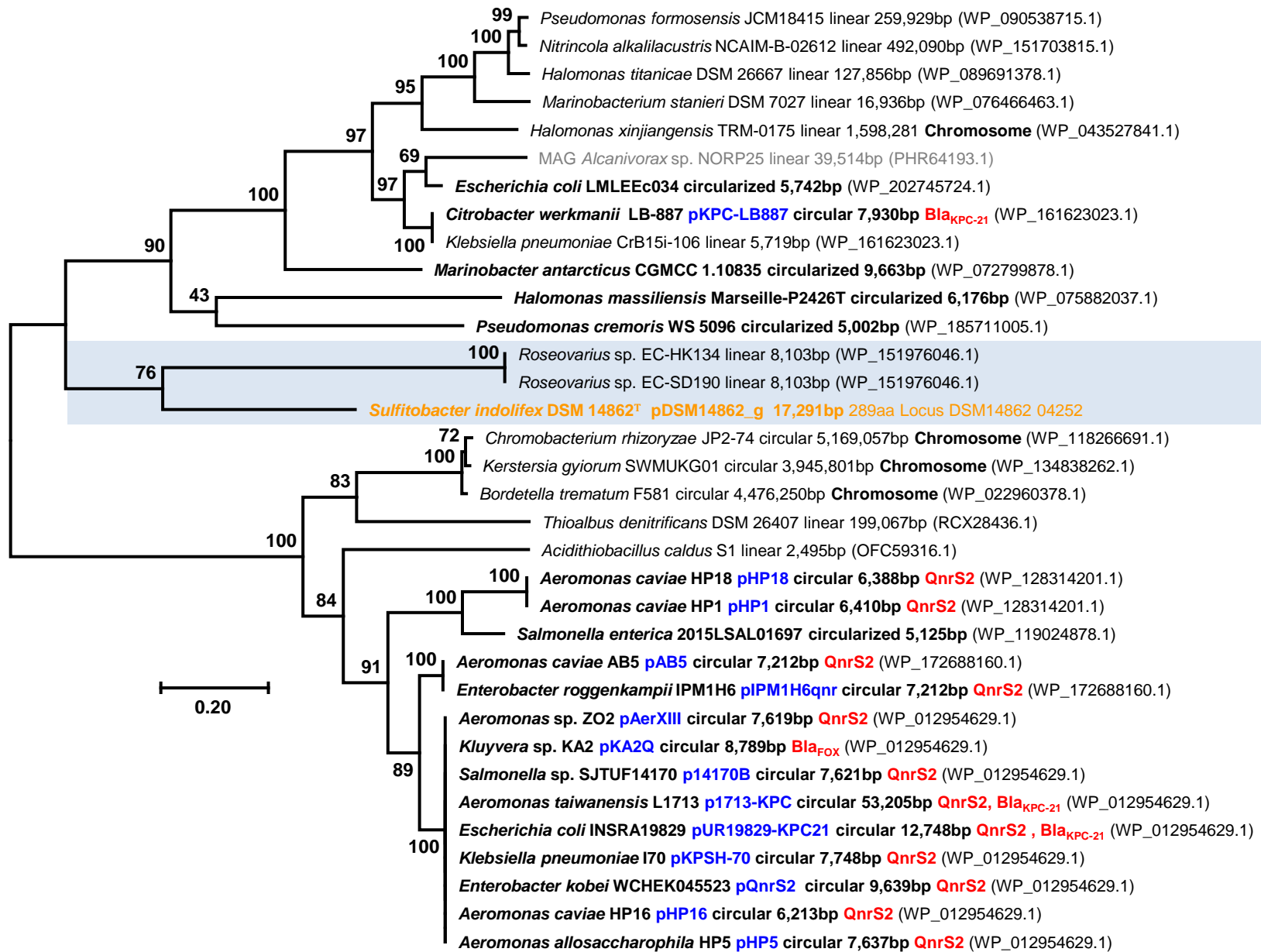

Figure S6

Supplement: Supplementary file 1 [file microorganisms-10-00738-s001.zip › Supplementary Figures & Tables/Supplementary Figures (PDF)/Figure_S6new_Phylogeny-Complete_RepQ_211010.pdf]

# Phylogenetic tree of RepW-type replicases

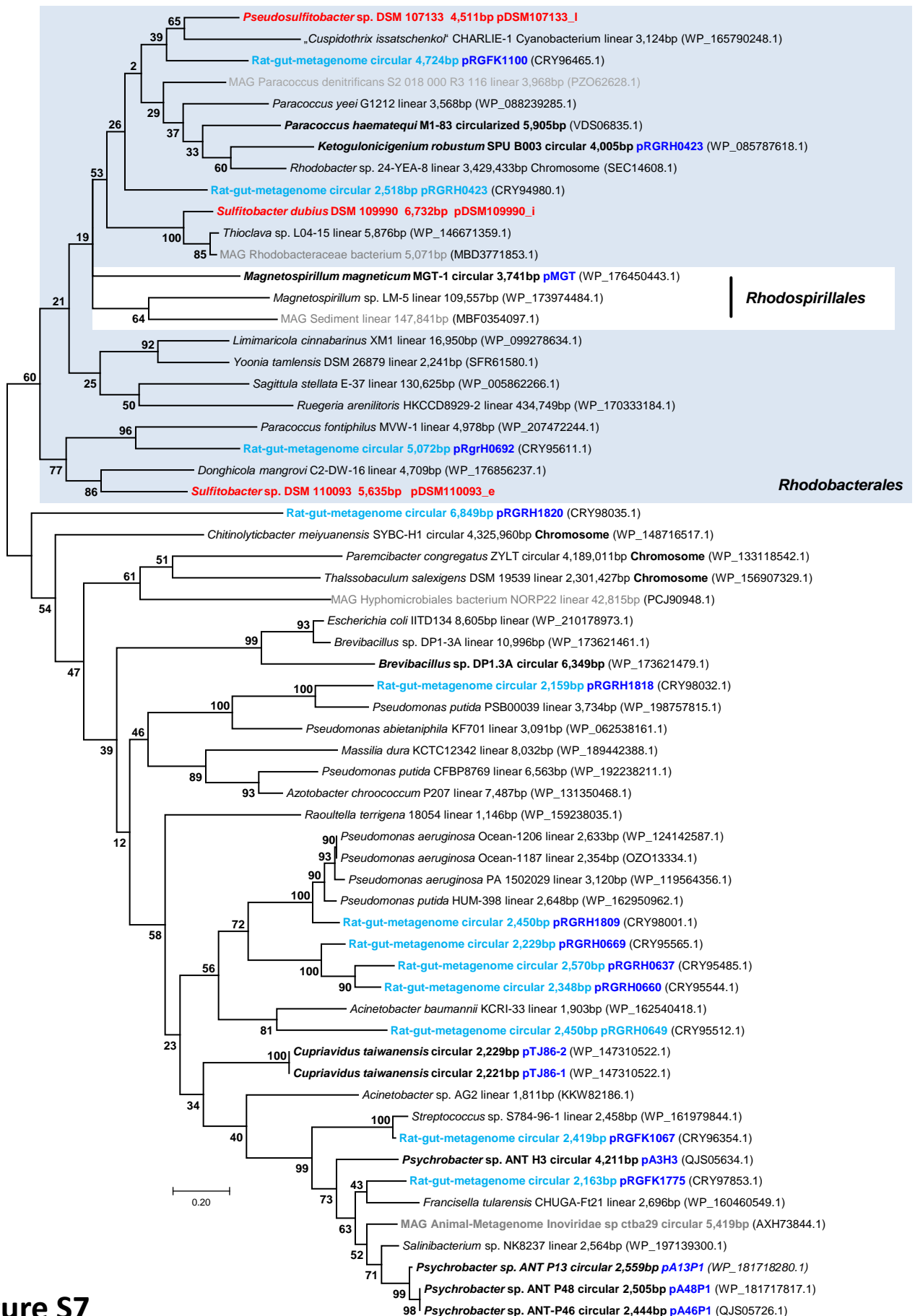

Figure S7

Supplement: Supplementary file 1 [file microorganisms-10-00738-s001.zip › Supplementary Figures & Tables/Supplementary Figures (PDF)/Figure_S7new_Phylogeny_RepW_61-Taxa_220225.pdf]
